# Prevalence and factors associated with depressive symptoms among patients with epilepsy in Ethiopia: a national-based systematic review and meta-analysis

**DOI:** 10.3389/fneur.2024.1352648

**Published:** 2024-02-28

**Authors:** Gebresilassie Tadesse, Techilo Tinsae, Girum Nakie, Gidey Rtbey, Fantahun Andualem, Asnake Tadesse, Mamaru Melkam, Girmaw Medfu Takelle, Setegn Fentahun

**Affiliations:** ^1^Department of Psychiatry, School of Medicine, College of Medicine and Health Sciences, University of Gondar, Gondar, Ethiopia; ^2^School of Nursing, College of Medicine and Health Sciences, University of Gondar, Gondar, Ethiopia

**Keywords:** depressive symptoms, epilepsy, systematic review, meta-analysis, Ethiopia

## Abstract

**Background:**

Depression is a major public health problem and negatively affects the quality of life of patients with epilepsy. Despite multiple studies investigating the magnitude and predictors, the results have been inconsistent. Therefore, this study aimed to estimate the pooled prevalence and factors associated with depressive symptoms among patients with epilepsy in Ethiopia.

**Methods:**

The primary articles were searched using databases like PubMed, Google Scholar, CINAHL, SCOPUS, EMBASE, and African Journal Online. A total of 10 primary articles that assessed the prevalence and factors associated with depressive symptoms among patients with epilepsy in Ethiopia were included. A Microsoft Excel spreadsheet was used to extract the data, which was then exported to Stata version 14 for further analysis. The statistical heterogeneity was evaluated using the *I*^2^ test. Due to heterogeneity, a random effect meta-analysis model was employed. Publication bias was checked through Egger’s weighted regression test and funnel plot.

**Results:**

A total of 10 primary studies with 3,532 participants were included. The pooled prevalence of depressive symptoms among patients with epilepsy was found to be 41.69% (95% CI, 37.70, 45.68). The pooled prevalence of depressive symptoms was 48.61, 42.98, 40.68, 38.27, and 34.80% in Oromia, SNNPs, Amhara, Addis Ababa, and Tigray, respectively, based on a sub-group analysis per regional state. Perceived stigma (AOR = 3.30, 95% CI: 1.40, 7.80), seizure frequency (AOR = 3.81, 95% CI: 1.03, 14.09), and perceived stress (AOR = 4.6, 95% CI: 1.05, 20.06) were factors associated with depressive symptoms.

**Conclusion:**

We found that depressive symptoms affects at least four out of ten patients with epilepsy, indicating an immense burden. Depressive symptoms were extremely prevalent in those who had high levels of stigma, a monthly seizure frequency of once or more, and perceived stress. Therefore, physicians should take extra precautions when treating patients with epilepsy who have certain conditions.

**Systematic review registration:**

This study was registered according to The International Prospective Register of Systemic Review (PROSPERO) with the registration ID (CRD42023484308).

## Introduction

The International League Against Epilepsy suggested that epilepsy be defined as a brain disease if it is either diagnosed as an epilepsy syndrome, if there are at least two unprovoked seizures that occur more than 24 h apart, or if there is one unprovoked seizure and a probability of additional seizures that is comparable to the general recurrence risk (at least 60%) after two unprovoked seizures (1).

Epilepsy is a neurological disease that affects people of all ages and affects both men and women. Even though epilepsy is more likely to be diagnosed in childhood and in the elderly, it is not limited to geography, socioeconomic class, race, and sex (2). According to the Global Burden of Epilepsy Report, 13 million disability-adjusted life years are attributed to epilepsy annually (3). Epilepsy affects over 70 million individuals worldwide, and 125,000 die each year, making it one of the most prevalent neurological conditions in the world (4). Of the 70 million people living with epilepsy, almost 85% of them live in low- and middle-income countries (4). Ethiopia has made significant improvements in important demographic and health indicators. Ethiopia is the second-most populous African nation, with a population of more than 110 million (5). Most individuals in countries like Ethiopia, where the majority of people are less aware of health issues, turn to traditional healers for relief from their illnesses, and traditional medicines are widely used in Ethiopia. Due to the healers’ cultural acceptance, traditional medicine’s low cost, and accessibility issues with modern healthcare facilities, up to 80% of individuals in developing countries use it (6). With the advancement of modern medicine over time, healthcare accounted for 5% of the country’s gross domestic product in 2014. According to the 2021–22 Ethiopian Ministry of Health annual performance report, a total of 17,534 health posts, 3,673 health centers, and 400 hospitals in the country were accessible. Public facilities make up 81.25% of all facility types. Both the overall number of hospitals and healthcare professionals are still inadequate (7). The incidence of epilepsy in Ethiopia was 29.5/1000 population (8), and it was estimated that 360,000 to 400,000 patients with epilepsy were living with poor medication (9). According to a recent meta-analysis, the estimated percentage of Ethiopians with poor knowledge of epilepsy was 48.54% (10), and almost 40% of patients with epilepsy were anti-seizure medication non-adherents (11). The overall poor treatment outcome (uncontrolled seizure) of patients with epilepsy in Ethiopia who received anti-seizure medications was 54% (12).

The presence of frequent seizures may hurt important aspects of life (at home, school, and the workplace), as well as occasionally impeding the formation of new interactions and friendships. If left untreated, epilepsy can lead to catastrophic brain injuries, physical impairments, decreased social interaction, and mental illness. It may cause considerable psychological distress as well as early death (13).

A significant number of studies suggest that patients with epilepsy in Ethiopia suffer from a range of mental health problems, the most prominent of which is depressive symptoms, which have a detrimental impact on their quality of life (14). It is possible for epilepsy and other comorbidities, like depression, to be related due to a shared etiology, shared genetic or environmental variables, or side effects from anti-seizure medications (15). Patients with epilepsy have an increased likelihood of experiencing depressive symptoms when compared with patients with chronic medical conditions or the general population (16, 17). The burden of depressive symptom among patients with epilepsy (PWE) is a worldwide problem with a range of 9–55% in developed countries, and the meta-analysis result shows 23.1% (18–20). Different studies conducted in Nigeria, Guinea, Kenya, and Rwanda revealed that the prevalence of depressive symptoms among PWE was found to be 85.5, 66, 16.5, and 14.2%, respectively (21–24). According to a meta-analysis and systematic review carried out in seven sub-Saharan countries, the prevalence of depressive symptoms among patients with epilepsy was 32.71% (25).

Studying the magnitude of comorbidity between depressive symptoms and epilepsy in Ethiopia has a significant role because of the high prevalence of epilepsy in this region. In Ethiopia, the magnitude of depressive symptoms among patients with epilepsy ranges from 32.8 to 51.2% (26, 27). For patients with epilepsy, having a depression-related comorbidity is linked to a poor quality of life and an increase in suicidal ideation (28, 29). Increased cognitive, emotional, and physical disease, as well as a markedly decreased overall seizure recovery, is linked to more severe comorbid depressive symptoms with epilepsy (30, 31). In addition, professionals may find it difficult to manage antidepressant medication for patients with comorbid depressive symptoms and epilepsy because of concerns about medication interactions, polytherapy adverse effects, and the potential to lower seizure thresholds (32).

Depressive symptoms in patients with epilepsy not only make treatment more difficult and lower quality of life, but also impose an additional burden on health systems, and there is a strong correlation between financial hardship and depressive symptoms. Patients with epilepsy who have untreated depressive symptoms typically utilize an overabundance of medical resources, especially in low-income nations (33). Patients with epilepsy who have mild to moderate depressive symptoms saw a two-fold increase in medical visits, while those with severe depressive symptoms saw a four-fold increase compared to patients with epilepsy without comorbid depressive symptoms (34).

There is evidence attributing several factors to a higher incidence of depressive symptoms in people with epilepsy. The frequency of seizures, age onset of a seizure, lower monthly income, adverse effects from anti-seizure medications, difficulties maintaining treatment, polytherapy, poor social support, and low educational status have all been identified as major risk factors for depression in Ethiopia (27, 35–39). Different literature at different times was collected and showed a high prevalence and inconclusiveness of the magnitude and predictors of depressive symptoms among patients with epilepsy in Ethiopia.

Although many primary studies were conducted, there is no evidence to conclude the pooled prevalence and significant factors of depressive symptoms among patients with epilepsy in Ethiopia. Therefore, this systematic review and meta-analysis aimed to assess the pooled prevalence and factors associated with depressive symptoms among patients with epilepsy in Ethiopia.

### Research questions

What is the estimated pooled prevalence of depressive symptoms among patients with epilepsy in Ethiopia?

What is the pooled effect of factors associated with depressive symptoms among patients with epilepsy in Ethiopia?

## Methods

### Protocol and registration of this study

The International Prospective Register of Systemic Review (PROSPERO) has the protocol for the current systematic review and meta-analysis registered (ID = CRD42023484308). We utilized the Preferred Reporting Items for Systematic Reviews and Meta-Analyses (PRISMA-2020) criteria, which are suitable guidelines for reports pertaining about systematic reviews and meta-analyses.

### Searching strategies and appraisal of studies

Using the PRISMA checklist criteria, desk reviews of doctoral dissertations, reference list reviews, and electronic web-based database searches were used to gather the original research and doctoral dissertation articles for this meta-analysis (40).

The primary studies included in this study were searched using the following databases: PubMed, HENARI, Psychiatry Online, Science Direct, SCOPUS, African Index Medicus (AIM), EMBASE, WHO’s Institutional Repository for Information Sharing (IRIS), CINAHL, and African Journals Online of the World Health Organization’s (WHO) database portal for low- and middle-income countries. Google Scholar and Google were also used to search for gray kinds of literature. In addition, the researchers found related articles through a desk review of the doctoral dissertations that are available at Ethiopian university libraries and institutional repositories, as well as by looking through the reference lists of related articles.

A search strategy was developed based on the prevalence of depressive symptoms and associated factors for each database by using a combination of free texts and controlled vocabularies such as Mesh terms and keywords. The following search items were used: “proportion” OR “magnitude” OR “epidemiology” OR “prevalence” OR “burden” OR “incident” AND “depressive symptoms” OR “depression” OR “major depressive disorder” OR “dysthymic disorder” OR “chronic depressive disorder” OR “depressive symptoms” AND “risk factors” OR “predictors” OR “determinants” OR “relationships” OR “correlates” OR “associated factors” AND “epileptic patients” OR “seizure” OR “patients with epilepsy.”

### Eligibility of the study

This systematic review and meta-analysis included all observational studies, like cross-sectional, case–control, and cohort study designs, on the prevalence and factors associated with depressive symptoms among patients with epilepsy in Ethiopia published before November 15, 2023. In this review, all articles with full text and easily available, written in the English language, conducted among patients with epilepsy were included. Nevertheless, this systematic review and meta-analysis did not include case reports, case studies, conferences, journals lacking entire articles, papers written in other languages, or qualitative findings.

### Data extraction

The titles, abstracts, and full texts of the included primary articles were carefully evaluated before the authors (SF and FA) separately extracted all the required data using a standard data extraction format organized in a Microsoft Excel spreadsheet. The final data extraction format contains the publication year, the first author’s name, the study design, the region where the study was conducted, the assessment tools used to screen for depressive symptoms, the sample size, and the prevalence of depressive symptoms. As the second objective, factors associated with depressive symptoms, including the 95% confidence interval and odds ratio, were also extracted. Any discrepancies between the two authors that arose throughout the data extraction process were resolved through discussion with the third author (GN), and double extraction of the data that was inconsistent was done to attain a common agreement. All primary articles conducted in Ethiopia and available online before November 15, 2023, were included and extracted.

### Measurement of the outcome variables

There are two primary objectives for this meta-analysis and systematic review. The first objective was to determine the pooled prevalence of depressive symptoms among patients with epilepsy in Ethiopia. Depressive symptoms were assessed through the Patient Health Questionnaire- Nine Items (PHQ-9), Beck’s Depression Inventory (BDI-II), and the Hospital Anxiety and Depression Scale (HADS). The PHQ-9 was validated among outpatients in Ethiopia (41). All things considered, the PHQ-9 items demonstrated with intra-class correlation coefficient of 0.92 and a Cronbach’s alpha of 0.81. The PHQ-9 threshold score of 10 provided the best discriminatory power for the clinical interview-based diagnosis of depressive symptoms. The Receiver Operating Characteristics (ROC) analysis indicated that sensitivity = 86% and specificity = 67%. It was validated in Ethiopia’s Oromia regional state in the Afan-Oromo language (42).

Additionally, HADS was employed in many studies and validated among Ethiopian cancer patients. The depression subscales showed an internal consistency of 0.76, according to Ayalu Aklilu Reda’s findings. For the depression subscales, the intra-class correlation coefficient (ICC) is 84% (43). Even though BDI-II was not validated in Ethiopia, many studies were carried out through it. It was validated among low-income African American medical outpatients. Reliability was established with good item-total inter-correlations and a high internal consistency of 0.9. Criterion-related validity was verified. According to confirmatory factor analysis, the BDI-II reflected two first-order factors (cognitive and somatic), which reflected a second-order section (depression). It was also validated among South African university students (44).

All three screening tools evaluated depressive symptoms in local languages based on the study population’s primary language. These tools were primarily validated in the English language and then translated to the local language of the study population by local language professional speakers. For example, the questionnaires used in the Amhara, Addis Ababa, and SNNPs regional states were in the Amharic language; the study carried out in the Tigray regional state was collected in the Tigrigna local language; whereas the study carried out in the Oromia regional state was collected through Afan-Oromo and Amharic. Two primary studies (Nigussie et al. and Addis et al.) assessed the perceived stigma through the Kilifi Stigma Scale (KSS). It had a good test–retest reliability of 0.92, internal consistency, and Cronbach’s α of 0.91. It was initially developed and validated in Kilifi, Kenya. It is a straightforward three-point Likert scale with scores of 0 as “not at all,” 1 as “sometimes,” and 2 as “always.” It comprises 15 elements, and the sum of the points for each item was used to determine the final score. The presence of perceived stigma was indicated when a score was higher than the value on the 66th percentile of the data (45).

Other studies (Tsegabrhan et al., Tegegne et al., and Chaka et al.) assessed the perceived stigma through three-item perceived stigma scales. It comprised dichotomous questions in which a positive response to any of the following three questions indicates perceived stigma (feeling that some people are uncomfortable with them, feeling that some people would prefer to avoid them, or feeling that people treat them like an inferior person). The overall possible score ranges from 0 = not felt stigma to 3 = maximally felt stigma. On the other hand, the frequency of seizures was reported based on the patient’s self-reports.

Perceived stress was also assessed through the Perceived Stress Scale (PSS). Each item is rated on a 5-point scale, ranging from never to almost always. Positively worded items are reverse scored, and the ratings are summed, with higher scores indicating more perceived stress. The cutoff value for the stress limit was set to ≥20. PSS had an internal consistency of Cronbach’s alpha for the total score of PSS = 0.79 (46).

Identifying the pooled effect size of variables associated with depressive symptoms among patients with epilepsy in Ethiopia was the second objective of this review. Employing STATA version 14.0, the pooled prevalence of depressive symptoms was calculated. Additionally, the pooled effect size of factors associated with depressive symptoms among patients with epilepsy was identified using the odds ratio with a 95% confidence interval.

### Quality assessment

Two authors (TT and GR) evaluated the quality of the primary studies included in this systematic review and meta-analysis using the standard critical appraisal tool. To assess the methodological quality of the prevalence of cross-sectional studies, the Joanna Briggs Institute (JBI) quality rating standards were initially developed (47).

Nine items ranging from 0 to 9 points (0–4 low, 5–7 moderate, and 8 and beyond high quality) make up this quality assessment tool. In the current systematic review and meta-analysis, articles scoring five or above were included. The third author (AT) arbitrated any disputes among the writers to reach a consensus regarding the quality assessment of the included articles.

### Data synthesis and analysis

For further analysis, the retrieved data from the Microsoft Excel spreadsheet was exported to STATA 14.0. Tables, forest plots, and texts are used to summarize and present the findings of this systematic review and meta-analysis. The *I*^2^ statistics test was used to determine whether there was statistical heterogeneity among the included studies (48). A random-effect meta-analysis model was used to evaluate the pooled effect size of all included studies at a 95% confidence interval because there was a high amount of heterogeneity in this review. Subgroup analysis was performed to determine the reason for heterogeneity using the assessment tools, publication year, and regional state. Sensitivity analysis was used to verify the findings of a single study on overall prevalence. The publication bias of the included papers was assessed using Egger weighted regression tests at a 5% significant level as well as a visual evaluation of the symmetry in the funnel plots (49, 50). Publication bias was defined in Egger’s test as occurring when the value of p was less than 0.05.

## Results

### Search results

**Figure 1:**
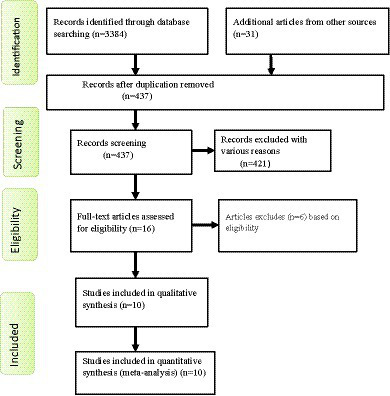
Flow chart shows study selection for a systematic review and meta-analysis of depressive symptoms among patients with epilepsy in Ethiopia.

For this review, a total of 3,415 articles were identified through a variety of electronic search methods, such as PubMed, Google Scholar, EMBASE, CINHAL, and African Journal Online. Among these studies, 2,978 articles were removed because of duplication. Furthermore, 421 studies which were irrelevant to our review, those not conducted in Ethiopia, had differences in study populations and settings and lacked complete texts were excluded after we assessed their titles and abstracts. Then, 16 full-text articles were reviewed for eligibility using the inclusion criteria, and 6 studies were excluded for other reasons. Finally, 10 studies were eligible and included in this systematic review and meta-analysis (Figure 1).

### Characteristics of the included studies

In this systematic review and meta-analysis study, a total of 10 primary studies that fulfilled the eligibility criteria were included. These 10 primary studies were carried out from October 2012 to February 2020 and published between July 7, 2014, and August 5, 2022. Regarding the regional states, three articles in Amhara (36, 38, 39), another three in SNNPs (26, 37, 51), two in Addis Ababa (27, 52), one study in Tigray (53), and another study in Oromia (35) were conducted.

A total of 3,532 patients with epilepsy were included in this review, with a sample size ranging from 114 (26) in SNNPS to 556 (38) in the Amhara regional state. Of the 10 included primary studies, only a single study revealed the magnitude of depressive symptoms in patients’ ages less than 18 years old (53). The other nine studies included adult patients with epilepsy. The age range of respondents ranged from 12 to 72 years. Out of 3,532 patients with epilepsy, 2,059 (58.3%) were males. Regarding the assessment tools, four studies (two studies in the Oromia, one study in the SNNPs, and the other study in the Amhara regional state) were conducted using the Beck Depression Inventory Scale (BDI-II) questionnaire. In contrast, three studies in Amhara and one study in Addis Ababa were assessed using the Hospital Anxiety and Depression Scale (HADS). On the other hand, three studies in SNNPs, Addis Ababa, and Tigray were assessed using a Patient Health Questionnaire (PHQ-9) to screen for the magnitude of depressive symptoms among patients with epilepsy in Ethiopia.

All primary articles included in this systematic review and meta-analysis was carried out using a cross-sectional study design. Based on the report from the included studies, patients with epilepsy in SNNPs (51.2%) and Addis Ababa (32.8%) were ranked as having the highest and least burden of depressive symptoms, respectively (Table 1).

**Table 1:** Characteristics of studies included in this systematic review and meta-analysis among patients with epilepsy in Ethiopia.

Authors	Assessment tools	Year of publication	Region	Sample size	Prevalence of depressive symptoms (%)	Number of anti-seizure medications	
						Monotherapy	Polytherapy
Tsegabrhan et al. (35)	BDI-II	2014	Oromia	300	49.3	153 (51%)	147 (49%)
Bifftu et al. (36)	BDI-II	2015	Amhara	405	45.2	309 (76.3%)	96 (23.7%)
Tegegne et al. (27)	HADS	2015	Addis Ababa	423	32.8	261 (61.9%)	162 (38.1%)
Angelo (51)	BDI-II	2018	SNNPs	244	51.2	217 (88.9%)	27 (11.1%)
Chaka et al. (52)	PHQ-9	2018	Addis Ababa	422	43.8	142 (33.6%)	280 (66.4%)
Duko et al. (26)	PHQ-9	2018	SNNPs	114	34.2	N/A	N/A
Engidaw et al. (37)	BDI-II	2020	Oromia	402	48.1	307 (76.4%)	95 (23.6)
Nigussie et al. (38)	HADS	2021	Amhara	556	39.9	439 (79%)	117 (21%)
Addis et al. (39)	HADS	2021	Amhara	370	37	244 (65.95%)	126 (34.05%)
Seid and Mebrahtu (53)	PHQ-9	2022	Tigray	296	34.8	216 (73%)	80 (27%)

SNNPs, Southern Nations Nationalities and Peoples; BDI-II, Beck’s Depression Inventory; HADS, Hospital Anxiety and Depression Scale; PHQ-9, Patient Health Questionnaire nine items.

### Clinical and medication related characteristics of patients with epilepsy in the included studies

Out of the 10 included studies, nine identified whether patients were under monotherapy or polytherapy. Concerning the distribution of the amount of medication they were taking, 2,280 (66.94%) out of 3,418 patients were taking one anti-seizure medication, whereas the remaining 1,130 (33.06%) patients were taking two or more anti-seizure medications (Table 1).

A study in Addis Ababa shows the duration of illness was more than 10 years in 36% of PWE, and 42.4% of patients had more than one seizure episode per month. From the total respondents, 59.5 and 66.4% of patients were diagnosed with grand-mal seizure and taking more than one anti-seizure medication, respectively (52). Based on the findings of the second study in Addis Ababa, 34.2% of patients stayed with epilepsy for more than 10 years, and most (54.9%) of patients had one or more seizure attacks per month (27).

A study carried out in the Benchi Maji zone of SNNPs shows nearly half (48.8%) of respondents had epilepsy for more than 11 years (51). The other study conducted in the Tigray regional state found that 55.1, 27.6, and 13.9% of patients had a seizure attack less than three times, three to five times, and six to ten times per month, respectively. The duration of treatment was more than 10 years in 29.7% of patients, while 82.1% of patients had improved on medication (53).

According to a study carried out in the Amhara regional state of the University of Gondar Hospital, the mean duration of the disease was 8.11 years, and 71.8% of patients had a seizure frequency of 1 to 11 times per year (36). The second study from this region revealed that 45.3 and 25.7% of patients were on anti-seizure medications for 1 to 6 and 7 to 12 years, respectively. The duration of the illness was up to 5 and 6 to 10 years among 62.8 and 21.8% of patients, respectively. Of those patients, 57.2 and 23.4% were taking phenobarbital and phenytoin, respectively (38). The third finding in the Amhara regional state (Addis et al.) shows 38.8, and 22.7% of patients stayed with epilepsy for six to ten and more than 11 years, respectively. 42.16% of patients attacked with a seizure episode for 1–3 times per year, while 28.8% of patients were attacked with more than once/month (39).

A study conducted in the Jimma hospital of the Oromia regional state found that 51.3% of patients had a seizure frequency of one to three times per month. The duration of epilepsy was between 1 and 5 years in about 40.3% of patients, while nearly one-third of patients lived with epilepsy between 6 and 10 years. Around 71% of patients were diagnosed with general tonic–clonic seizures (35). The other study in the Ilu Ababore zone of the Oromia regional state found that 35.6, 29.6, and 24.6% of patients stayed with epilepsy for the duration of 2 to 5 years, 6 to 10 years, and 11 and above years, respectively. The treatment duration was 6 years or less for the majority (54.7%), while the remaining (45.3%) of PWE were on treatment with anti-seizure medications for more than 6 years. The seizure frequency was 1 to 11 times per year for 71.6% of patients, and 94.52% of patients were under phenobarbital (37).

### The pooled prevalence of depressive symptoms among patients with epilepsy in Ethiopia

**Figure 2:**
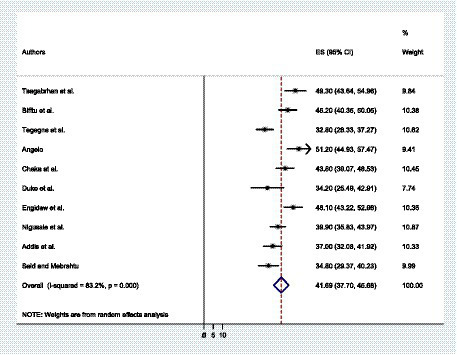
The forest plot shows the pooled prevalence of depressive symptoms among patients with epilepsy in Ethiopia.

A total of 10 primary studies were included to determine the prevalence of depressive symptoms among patients with epilepsy in Ethiopia. The overall prevalence of depressive symptoms among patients with epilepsy in Ethiopia was found to be 41.69% with a 95% CI (37.70, 45.68) (Figure 2). The weighted prevalence of depressive symptoms was also identified.

### Heterogeneity and publication bias

**Figure 3:**
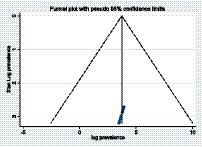
A funnel plot shows the pooled prevalence of depressive symptoms among patients with epilepsy in Ethiopia.

The statistics test (*I*^2^) was employed to assess the statistical heterogeneity, and there was a high level of heterogeneity in this study (*I*^2^ = 83.2%, value of *p* = 0.001). To identify publication bias in the included studies, two techniques were used: The first was checked using a funnel plot, which showed the symmetric distribution and revealed the absence of publication bias in the included articles (Figure 3). The second technique was the Eggers test, which revealed no publication bias in these studies, as evidenced by *p* = 0.722 (Table 2).

**Table 2:** Egger’s test of depressive symptoms among patients with epilepsy in Ethiopia.

Std-Eff	Coef.	Std. Err.	*T*	P > t	95% Conf. Interval
Slope	37.13415	11.93033	3.11	0.014	9.622769 64.64553
Bias	1.690854	4.585454	0.37	0.722	0.883222 12.26493

### Sub-group analysis

Since heterogeneity affected the pooled prevalence of depressive symptoms, we undertook a sub-group analysis according to the year of publication, the assessment tools, and the regional state. Based on a sub-group analysis per regional state, the pooled prevalence of depressive symptoms among patients with epilepsy was found to be higher in Oromia at 48.61% (44.91, 52.31). It was found that the overall prevalence of depressive symptoms among epileptic patients in the Amhara regional state and the SNNPs was 40.68% (36.22, 45.14) and 42.98% (26.33, 59.63), respectively. On the other hand, Addis Ababa had the lowest prevalence, at 38.27% (27.49, 49.05). Compared to studies that evaluated depressive symptoms using PHQ-9 and HADS, the overall prevalence of depressive symptoms among epileptic patients using BDI-TM was found to be greater. The pooled prevalence of depressive symptoms among epileptic patients in studies conducted before 2020 was found to be greater than that of studies conducted during and after 2020, with respective rates of 42.87% (36.84, 48.91) and 40.01% (34.56, 45.46) (Table 3).

**Table 3:** Sub-group analysis of depressive symptoms among patients with epilepsy in Ethiopia.

Variables	Sub-groups	Number of studies	Prevalence (95%CI)	*I*^2^ (%)	*p*-value
Region	Addis Ababa	2	38.27 (27.49, 49.05)	90.9	0.001
	Amhara	3	40.68 (36.22, 45.14)	64.5	0.060
	SNNPs	2	42.98 (26.33, 59.63)	80.4	0.006
	Oromia	2	48.61 (44.91, 52.31)	92.4	0.000
Assessment tools	BDI-II	4	48.05 (45.39, 50.1)	0.0	0.478
	HADS	3	36.63 (32.42, 40.84)	62.3	0.070
	PHQ-9	3	38.06 (31.32, 44.80)	73.0	0.025
Year of publication	<2020	6	42.87 (36.84, 48.91)	86.1	0.000
	≥2020	4	40.01 (34.56, 45.46)	80.6	0.001

SNNPs, Southern Nations Nationalities and Peoples; BDI-II, Beck’s Depression Inventory; HADS, Hospital Anxiety and Depression Scale; PHQ-9, Patient Health Questionnaire nine items.

### Sensitivity analysis of the study

In this systematic review and meta-analysis, the sensitivity analysis was carried out to examine the heterogeneity of those studies systematically by excluding one study to determine the impact of each study’s findings on the pooled prevalence of depressive symptoms. After we applied the sensitivity analysis, all of the values fell within the expected 95% CI, providing that the exclusion of a single study did not significantly alter the prevalence of this review (Table 4).

**Table 4:** Sensitivity analysis of depressive symptoms among patients with epilepsy in Ethiopia.

Authors	Estimated 95%CI	Heterogeneity	
		*I* ^2^	*p*-value
Tsegabrhan et al.	40.86 (36.78, 44.95)	82.4%	≤0.001
Bifftu et al.	41.28 (36.89, 45.67)	84.3%	≤0.001
Tegegne et al.	42.77 (38.98, 46.55)	78.4%	≤0.001
Angelo	40.71 (36.75, 44.67)	81.7%	≤0.001
Chaka et al.	41.44 (36.97, 45.91)	84.8%	≤0.001
Duko et al.	42.32 (38.16, 46.48)	84.2%	≤0.001
Engidaw et al.	40.95 (36.80, 45.10)	82.4%	≤0.001
Nigussie et al.	41.90 (37.32, 46.48)	84.95	≤0.001
Addis et al.	42.23 (37.89, 46.57)	84.0%	≤0.001
Seid and Mebrahtu	42.46 (38.28, 46.63)	83.0%	≤0.001

### Factors associated with depressive symptoms among patients with epilepsy in Ethiopia

From the included primary studies in this systematic review and meta-analysis, there are different factors significantly associated with depressive symptoms among patients with epilepsy in Ethiopia. Perceived stigma and the frequency of seizures once or more per month are significantly associated with depressive symptoms in five and four studies, respectively. Polypharmacy, difficulty with medication adherence, and onset of illness before 6 years old were associated with depressive symptoms in three studies. Furthermore, perceived stress in two studies was one of the factors significantly associated with depressive symptoms among patients with epilepsy in the primary articles that were included in this systematic review and meta-analysis. In this systematic review and meta-analysis, high perceived stigma, frequency of seizure once or more per month, difficulty for medication adherence, polypharmacy, onset of illness before 6 years old, and high perceived stress are factors significantly associated with depressive symptoms among patients with epilepsy. Based on our findings, the weighted odds ratio revealed that patients with epilepsy who have high perceived stigma were 3.36 times more likely to have depressive symptoms when compared to patients without perceived stigma (AOR = 3.36, 95% CI: 2.20, 5.14). Patients who used two or more pharmaceutical medications (Polypharmacy) were 2.09 times more prone to depressive symptoms than monotherapy (AOR = 1.54, 2.86). The likelihood of experiencing depressive symptoms was 3.55 times more odds among patients with epilepsy whose onset of illness was before 6 years of age compared to patients with onset of illness after 6 years of age (AOR = 3.55, 95% CI: 1.64, 7.70). Those patients with epilepsy who had difficulties of adhering their anti-seizure medications were 3.85 times (AOR = 95% CI: 2.69, 5.52) more likely to develop depressive symptoms than patients adhering medications regularly. Patients with epilepsy who were experienced with having a seizure frequency of once or more per month were 3.84 times more vulnerable to depressive symptoms compared to their counterparts (AOR = 3.84, 95% CI: 2.34, 6.31). In this study, participants who have high perceived stress have 4.51 times higher odds of having depressive symptoms than patients with epilepsy without having perceived stress (AOR = 4.51, 95%CI: 2.36, 8.61).

## Discussion

A total of 3,532 patients with epilepsy were included in this meta-analysis and systematic review. This study aimed to estimate the cumulative prevalence of depressive symptoms and its contributing variables among Ethiopian patients with epilepsy. Additionally, it is the first systematic review and meta-analysis of patients with epilepsy conducted in Ethiopia that provides aggregated information supporting the hypothesis that these populations—which are particularly exposed—have a higher prevalence of depressive symptoms. This review also identifies the significance of the burden in different dimensions, like regional state, assessment tools for the outcome variable, and years of publication.

Studies on depressive symptoms with epilepsy were more numerous than those on epilepsy with depression. Many studies focus on depressive symptoms with epilepsy; this may be because depressive symptoms predicts a worse response to treatment during epilepsy (54) and because individuals with depressive symptoms have a higher risk of suicide (55). This correlation could be causative, or epilepsy and depressive symptoms could have similar pathogenic pathways. Depressive symptoms is becoming more common in people with epilepsy (56). We found that the burden of depressive symptoms was higher among epileptic patients. The pooled prevalence of depressive symptoms among patients with epilepsy in Ethiopia was found to be 41.69% with a 95% CI (37.70, 45.68). In this systematic review and meta-analysis, the pooled prevalence of depressive symptoms was higher than the findings of another study that included seven sub-Saharan countries (32.7%) (25). The difference in the assessment instruments could be the cause of the discrepancy. For example, most of the primary studies included in the above comparative study used the Mini International Neuropsychiatric Interview (MINI), Diagnostic Interview Schedule for Children version IV (DISC-IV), and Brief Psychiatric Rating Scale (BPRS). In this review, most studies used HADS, PHQ-9, and BDI-II. Therefore, the difference in the assessment tools might overestimate the magnitude of depressive symptoms in our findings. This finding also had a significantly higher magnitude than other studies carried out in China (34%) (57), Australia (22.9%) (58) and America (23.1%) (18). The high prevalence of depressive symptoms reported in the studies under review suggests that the psychological toll of epilepsy may be particularly severe in low-income settings (59, 60). In Ethiopia, the illness is poorly managed, treatment options are limited, there is high epilepsy-related stigma, and the social and economic costs of illness are particularly extreme. Despite this, we still expected to find significant comorbidity due to the well-established bi-directional relationship between depressive symptoms and epilepsy. In addition to being neurological, socioeconomic variables also influenced the association between depressive symptoms and epilepsy (61). More so than physical considerations, the psychosocial functioning of the patients has a significant impact on their quality of life. The unfavorable social context in our patient’s encounter may have a greater psychological and social influence on QOL than physical issues, leading to high degrees of despair and social unhappiness. It is often known that epilepsy is stigmatized in our country, and from the standpoint of the patient, this is still a significant problem that has to be addressed (62, 63).

Because we have observed heterogeneity, we applied a sub-group analysis based on assessment tools, regional states, and year of publication. The findings of depressive symptoms using the BDI-II were found to be higher than studies that were assessed using the PHQ-9 and HADS. The pooled prevalence of depressive symptoms among patients with epilepsy was found to be higher in the Oromia regional state at 48.61% (44.91, 52.31). On the other hand, the pooled prevalence of depressive symptoms among patients with epilepsy in the SNNPs and Amhara regional states was found to be 42.98% (26.33, 59.63), 40.68% (36.22, 45.14), respectively. In contrast, the lowest prevalence was found in Addis Ababa: 38.27% (27.49, 49.05). The pooled prevalence of depressive symptoms among patients with epilepsy that was conducted before 2020 was higher than studies carried out during and after 2020: 42.87% (36.84, 48.91) and 40.01% (34.56, 45.46), respectively (Table 3).

**Figure 4:**
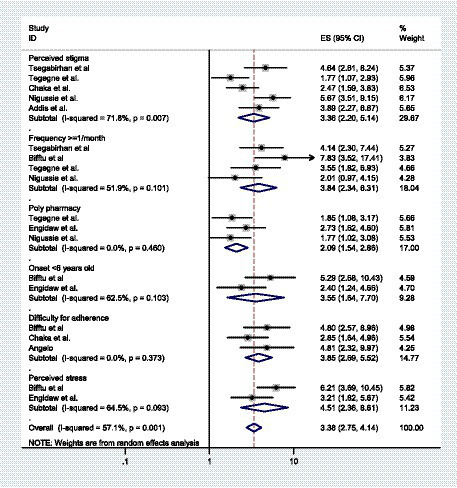
A forest plot shows factors significantly associated with depressive symptoms among patients with epilepsy in Ethiopia.

Based on the literature, risk factors for depressive symptoms in patients with epilepsy have been identified to investigate the variables affecting the association between depressive symptoms and epilepsy. Eight papers in this analysis reported the associated factors of depressive symptoms among patients with epilepsy. From the reviewed articles, high perceived stigma, polypharmacy, seizure frequency, difficulty in adherence, onset of illness before 6 years old and perceived stress were factors significantly associated with depressive symptoms in patients with epilepsy. This study found that all reviewed factors, like high perceived stigma, polypharmacy, frequency of seizure once or more per month, difficulty in adherence, onset of illness before 6 years old, and high perceived stress, were factors associated with depressive symptoms among PWE (Figure 4).

Accordingly, PWE who had high perceived stigma were more than three times more likely to develop depressive symptoms compared to patients without perceived stigma (AOR = 3.36, 95% CI: 2.20, 5.14). This finding was consistent with a study carried out in Kenya (64) and China (65). The social prognosis of epilepsy is significantly affected by stigma, which can prevent individuals from obtaining treatment and have negative effects on quality of life and social inclusion (66). According to a meta-analysis (67), there is a positive correlation between perceived stigma and disease-related features such as “seizure severity,” “seizure frequency,” “number of medications,” and “adverse events.” Perceived stigma was strongly correlated with depressive symptoms, as seen by multiple studies showing that depression levels increased in line with stigma levels (68). Seizures without control can be extremely disturbing. People might even be afraid to leave their houses alone. If they were to have a seizure in public, they might be afraid of what other people would think of them. Epilepsy has historically and globally been a socially stigmatized condition. People who experience such devaluation are frequently stigmatized and are subjected to psychological hardships and mental illnesses like depression (69).

Patients who used two or more anti-seizure medications were two times more prone to depressive symptoms than those on monotherapy (AOR = 1.54, 2.86). Other findings from a systematic review and meta-analysis also showed this positive association (25). Evidence indicates that polytherapy raises the number of prescription medications taken in addition to providing poor medication adherence, which enhances the risk and severity of adverse reactions to drugs due to inadequate seizure control (70). The burden and adverse consequences of anti-seizure medications could be the cause (71). Studies have shown that certain barbiturates, particularly phenobarbital, have been associated with a higher incidence of depressive symptoms. Additionally, those undergoing polytherapy may be more prone to committing medication errors and have an elevated likelihood of drug-to-drug interactions (70). Our findings suggest that adequate medication therapy should receive more attention, and pill burden, tolerability, and potential drug interactions should be carefully considered.

The likelihood of experiencing depressive symptoms was 3.55 times higher among patients with epilepsy whose onset of illness was before 6 years of age compared to patients with onset of illness after 6 years of age (AOR = 3.55, 95% CI: 1.64, 7.70). According to different studies, epilepsy imposes a significant strain on the family, the community, and society at large. These pressures showed up in a variety of areas of their lives, including their financial difficulties (72, 73), psychological well-being (74, 75), and physical health (76). Additionally, it may be related to the fact that the study participants lacked sufficient stress management techniques for the issues mentioned above. This is due to the possibility that in their early years, they lacked the knowledge and experience necessary to deal with the stigma, disease, and various cultural beliefs that led to the development of comorbid psychiatric disorders.

Those patients who had difficulties adhering to their anti-seizure medications were 3.85 times (AOR = 95% CI: 2.69, 5.52) more likely to develop depressive symptoms than patients adhering to medications regularly. One explanation could be that individuals who hesitate to take their anti-seizure medications as prescribed could have breakthrough seizures, and medication discontinuations and withdrawals can result in a recurrence of seizure episodes. The majority of studies indicate that, compared to individuals without seizures, those with uncontrolled seizures have a higher prevalence of depressive symptoms (77).

This systematic review and meta-analysis showed that epileptic patients with a seizure frequency of once or more per month had nearly four times higher odds of experiencing depressive symptoms than those who had controlled seizure (AOR = 3.84, 95% CI: 2.34, 6.31). This finding was supported by another studies conducted in Guinea (22) and Sri Lanka (78). This could bring on fear and uncertainty about the next seizure, which could then result in feelings of hopelessness and worthlessness. The fact that epilepsy’s symptoms and indicators are unusually obvious, erratic, and difficult to comprehend could be the cause (79). The inability to pinpoint the location and timing of a seizure may be linked to socially unacceptable symptoms such as loose stool, urination, tongue biting, and foaming at the mouth, which can cause epileptic individuals to experience a variety of psycho-social issues and mental health-related problems, including depressive symptoms (77).

Furthermore, patients with epilepsy who had high perceived stress had more than four times higher odds of experiencing depressive symptoms compared with patients with low perceived stress (AOR = 4.51, 95% CI: 2.36, 8.61). Our finding was consistent with a study undertaken in England (80). This could be because people who experience high levels of perceived stress may find it more difficult to adjust psychologically to various events in their lives, such as perceived stigma, unemployment, low educational attainment, and frequent seizures, which could lead to comorbid psychiatric illness. According to a study, the most common types of stressors that appear to be associated with depressive symptoms are those that impair a person’s sense of self-worth, those that cause one to become frustrated about achieving a goal, and any stressor of inappropriate intensity (81). Additionally, people who experience high levels of perceived stress may find it more difficult to adjust psychologically to various stressful life events, such as losing a major job, suffering a serious illness, or receiving a new diagnosis. Numerous studies have also demonstrated that stress can result in brain neurotransmitter imbalances, including serotonin, which can cause depressive symptoms (82, 83).

### Strengths and limitations of the study

To reduce reviewers’ biases and the evaluation of the papers’ quality by reviewers, the authors of this study employed research across many databases. Showing the estimated pooled prevalence and the pooled related factors, as well as performing subgroup analysis using the regional states as well as assessment tools.

### Limitations of the study

Despite the numerous advantages of this systematic review and meta-analysis, the combined effect of depression among patients with epilepsy has the following limitations: all of the primary studies included in this review were carried out using a cross-sectional study design, which only illustrates a temporal relationship rather than a true cause-and-effect relationship. So the cause and effect of depressive symptoms in patients with epilepsy is not well defined and elucidated in this study.

A small number of studies were included in the systematic review and meta-analysis, and there was heterogeneity among the primary articles that were included in the review. Even though we have employed sub-group analysis, there are no established guidelines for the diagnosis or screening of depressive symptoms. For instance, the Beck Depression Inventory (BDI), the Hospital Anxiety and Depression Scale (HADS-7), and the 9-item Patient Health Questionnaire (PHQ-9) were frequently used in the included articles. However, it should be noted that evaluation based solely on those three questionnaires, rather than clinical evaluations, tend to introduce bias and overestimate the prevalence. Some variables, like frequency of seizure, age at onset of illness, and medication adherence problems, were assessed without standard tools, which might affect the overall inference of factors to depressive symptoms.

### Implications of this finding

Future researchers should take note of this data, as it indicates an increased prevalence of depression among individuals with epilepsy. It also has implications for physicians and policymakers. Consequently, more research is required to determine the cause of an increase in depression and to provide more effective treatment. When epileptic patients appear in the community or at the hospital, clinicians ought to evaluate them for depression. With the help of this study, policymakers should be able to create more effective preventative and treatment plans in the community and healthcare facilities.

## Conclusion and recommendation

This systematic review and meta-analysis found that at least four in ten epileptic patients have depression. This indicated that the overall burden of depression among patients with epilepsy was high. Our finding concluded that high perceived stigma, frequency of seizures, and high perceived stress were some of the factors significantly associated with depression. The sub-group analysis based on region, assessment tools, and year of publication showed that there was also a significant difference in the pooled prevalence of depressive symptoms among patients with epilepsy. The findings of this study would be used as baseline data to create more manageable and preventive strategies for those with comorbid depressive symptoms and epilepsy at the country level. This study was also helpful to others who might be looking to learn more about the comorbidity between depressive symptoms and epilepsy. It identified the potential predictors of depressive symptoms for policymakers and those who work in the area of neuropsychiatric setups to easily design the prevention and management mechanisms. Creating public awareness interventions, policy-based interventions, school-based interventions, and interventions that target patients with epilepsy themselves, as well as their caregivers and peers, could decrease the perceived stigma and stress of patients with epilepsy. Regular screening of patients with epilepsy by well-trained mental health professionals is recommended for the early management and treatment of depressive symptoms.

## Data availability statement

The original contributions presented in the study are included in the article/Supplementary material, further inquiries can be directed to the corresponding author.

## Author contributions

GT: Conceptualization, Data curation, Formal analysis, Investigation, Methodology, Software, Writing – original draft, Writing – review & editing. TT: Data curation, Supervision, Writing – review & editing. GN: Data curation, Resources, Writing – review & editing. GR: Resources, Writing – original draft. FA: Formal analysis, Writing – original draft. AT: Investigation, Writing – review & editing. MM: Supervision, Writing – original draft. GMT: Writing – review & editing, Resources. SF: Methodology, Software, Writing – original draft.
