# Supplementary material for: Prevalence and factors associated with depressive symptoms among patients with epilepsy in Ethiopia: a national-based systematic review and meta-analysis
Source: Front Neurol. 2024 Feb 28;15:1352648. doi: 10.3389/fneur.2024.1352648 (PMC10946423; doi:10.3389/fneur.2024.1352648)
Supplement: Supplementary file 2 [file Table_2.docx]

Table_S2 Quality assessment of depression and associated factors among epileptic patients in Ethiopia that were included studies in this meta-analysis and systematic review.

| Authors, and year of  Publication | Q1 | Q2 | Q3 | Q4 | Q5 | Q6 | Q7 | Q8 | Q9 | Total score |
| --- | --- | --- | --- | --- | --- | --- | --- | --- | --- | --- |
| Tsegabrhan et al. 2014 | Y | Y | Y | Y | Y | Y | Y | Y | Y | 9 |
| Biﬀtu et al. 2015 | Y | Y | Y | NA | Y | Y | Y | NA | Y | 7 |
| Tegegne et al. 2015 | Y | Y | Y | Y | Y | Y | Y | Y | Y | 9 |
| Angelo 2018 | Y | Y | NA | Y | NA | Y | Y | Y | Y | 7 |
| Chaka et al. 2018 | Y | Y | Y | Y | Y | Y | Y | Y | Y | 9 |
| Duko et al. 2018 | Y | Y | NA | Y | Y | Y | Y | NA | Y | 7 |
| Engidaw et al. 2020 | Y | Y | Y | Y | Y | Y | Y | Y | Y | 9 |
| Nigussie et al. 2021 | Y | Y | NA | Y | Y | Y | NA | Y | Y | 7 |
| Addis et al. 2021 | Y | Y | Y | Y | Y | Y | Y | Y | Y | 9 |
| Seid and Mebrahtu 2022 | Y | NA | Y | Y | Y | Y | NR | Y | Y | 7 |

**Key:** **Y**= Yes; **NR**= Not reported, **NA**=Not appropriate

Question codes:

1. Was the sample frame appropriate to address the target population?

2. Were study participants sampled in an appropriate way?

3. Was the sample size adequate?

4. Were the study subjects and the setting described in detail?

5. Was the data analysis conducted with sufficient coverage of the identified sample?

6. Were valid methods used for the identification of the condition?

7. Was the condition measured in a standard, reliable way for all participants?

8. Was there appropriate statistical analysis?

9. Was the response rate adequate, and if not, was the low response rate managed appropriately?
